# Factors affecting uptake and adherence to breast cancer chemoprevention: a systematic review and meta-analysis

**DOI:** 10.1093/annonc/mdv590

**Published:** 2015-12-08

**Authors:** S. G. Smith, I. Sestak, A. Forster, A. Partridge, L. Side, M. S. Wolf, R. Horne, J. Wardle, J. Cuzick

**Affiliations:** 1 Wolfson Institute of Preventive Medicine, Queen Mary University of London, London; 2 Health Behaviour Research Centre, University College London, London, UK; 3 Department of Medical Oncology, Dana-Farber Cancer Institute, Boston, USA; 4 Institute for Women's Health, University College London, London, UK; 5 Division of General Internal Medicine, Northwestern University, Chicago, USA; 6 Centre for Behavioural Medicine, University College London, London, UK

**Keywords:** preventive therapy, chemoprevention, decision-making, adherence, uptake, medication

## Abstract

**Background:**

Preventive therapy is a risk reduction option for women who have an increased risk of breast cancer. The effectiveness of preventive therapy to reduce breast cancer incidence depends on adequate levels of uptake and adherence to therapy. We aimed to systematically review articles reporting uptake and adherence to therapeutic agents to prevent breast cancer among women at increased risk, and identify the psychological, clinical and demographic factors affecting these outcomes.

**Design:**

Searches were carried out in PubMed, CINAHL, EMBASE and PsychInfo, yielding 3851 unique articles. Title, abstract and full text screening left 53 articles, and a further 4 studies were identified from reference lists, giving a total of 57. This review was prospectively registered with PROSPERO (CRD42014014957).

**Results:**

Twenty-four articles reporting 26 studies of uptake in 21 423 women were included in a meta-analysis. The pooled uptake estimate was 16.3% [95% confidence interval (CI) 13.6–19.0], with high heterogeneity (*I*^2^ = 98.9%, *P* < 0.001). Uptake was unaffected by study location or agent, but was significantly higher in trials [25.2% (95% CI 18.3–32.2)] than in non-trial settings [8.7% (95% CI 6.8–10.9)] (*P* < 0.001). Factors associated with higher uptake included having an abnormal biopsy, a physician recommendation, higher objective risk, fewer side-effect or trial concerns, and older age. Adherence (day-to-day use or persistence) over the first year was adequate. However, only one study reported a persistence of ≥80% by 5 years. Factors associated with lower adherence included allocation to tamoxifen (versus placebo or raloxifene), depression, smoking and older age. Risk of breast cancer was discussed in all qualitative studies.

**Conclusion:**

Uptake of therapeutic agents for the prevention of breast cancer is low, and long-term persistence is often insufficient for women to experience the full preventive effect. Uptake is higher in trials, suggesting further work should focus on implementing preventive therapy within routine care.

## Abstract (precis)

In this systematic review of studies investigating decision-making in the context of breast cancer preventive therapy, we observed low uptake of all agents and poor long-term persistence. Our meta-analysis including over 21 000 women demonstrated that only 1 in 6 eligible women decided to take preventive therapy. Persistence for 5 years was low, limiting the preventive effect in these women.

## introduction

Breast cancer is the most commonly diagnosed cancer in women, with an estimated 1.67 million new cases diagnosed worldwide in 2012 [1]. Over 500 000 deaths are recorded each year, making it the leading cause of cancer death in women [1]. It is expected that one in eight US women will be diagnosed with the disease in their lifetime [2]. A decline in breast cancer mortality has been observed over the last 40 years [3, 4], although incidence continues to rise [5, 6], particularly in developing countries [7]. A number of factors have been associated with an increased risk of developing breast cancer [8], including family history which accounts for ∼5%–10% of all breast cancers.

Preventive therapy is a risk reduction option for women who have an increased risk of breast cancer. Selective Estrogen Receptor Modulators (SERMs) have been extensively tested, and trials of alternative agents are ongoing. A meta-analysis of 10-year individual-level data from nine randomized SERM trials demonstrated a 38% reduction in overall breast cancer incidence and a 51% reduction in estrogen receptor positive (ER+) tumours [9]. The preventive effect of tamoxifen can last at least 20 years [10]. Women taking SERMs have more venous thromboembolic events and more endometrial cancers [9]. Menopausal symptoms such as hot flashes and vaginal dryness are also more common among women taking SERMs, which can affect tolerability [11].

The effectiveness of preventive therapy to reduce breast cancer incidence at a population level depends on adequate levels of uptake and adherence to therapy. The discovery and testing of new agents also relies on acceptability to the population. An estimated 2 million US women and 500 000 UK women have favourable cost–benefit profiles for the prophylactic use of tamoxifen [12, 13]. However, a meta-analysis of five studies reporting uptake data in non-trial settings found a mean uptake of just 14.8% among women offered the opportunity to take preventive therapy [14]. Trial data were not included in this review. Independent studies and narrative reviews have also raised concern about the low levels of long-term adherence to preventive therapy [11, 15, 16], but no systematic synthesis has been done.

To make recommendations for future research and clinical practice, this review aims to synthesize the available quantitative data on uptake of preventive therapy and adherence among women who have an increased risk of breast cancer in either trial or non-trial settings. To aid the development of behavioural interventions, we aimed to identify the sociodemographic, clinical and psychological factors associated with uptake and adherence. Qualitative studies were also included in this investigation to supplement our understanding of women's decision-making in this context.

## methods

### search strategy

We searched for quantitative articles reporting uptake and adherence to medications used for the purpose of preventing primary breast cancer, and quantitative and qualitative articles reporting factors affecting these decisions. Adherence included either adequate day-to-day use of the medication or persistence with it over time. In November 2014, separate searches were carried out in PubMed, CINAHL, EMBASE and PsychInfo (see supplementary Appendix S1, available at *Annals of Oncology* online for example search terms). The review was prospectively registered on the PROSPERO database [17] (registration number: CRD42014014957). PRISMA guidelines were followed throughout [18] (supplementary Appendix S2, available at *Annals of Oncology* online).

### article selection

The inclusion criteria were peer-reviewed studies: in English language; including women aged 18 years or older; reporting quantitative or qualitative data; including at least one aspect of medication use (uptake, day-to-day adherence with prescription guidelines and/or persistence with the medication over time); and using or testing the agent for the purpose of breast cancer prevention. Qualitative studies had to investigate eligible women's perceptions of preventive therapy and explanations for their decisions associated with chemoprevention. The exclusion criteria were studies including women affected by breast cancer (including ductal carcinoma *in situ*), agents where the primary purpose was not breast cancer prevention, hypothetical rates of adherence, men only, clinician perspectives, non-peer-reviewed studies, conference abstracts, reviews, interventions not involving oral agents and commentaries and letters not including empirical data. No restriction was placed on publication dates or study design.

After removing duplicates, two authors (SGS, AF) used the inclusion and exclusion criteria to review half of the titles and abstracts each. The same authors checked the excluded articles of the other person to ensure sensitivity. A similar process was undertaken for the full texts. The remaining article's reference lists were examined to identify studies not included in our search. The articles included in the meta-analysis were decided by mutual discussion (SGS, IS).

### data extraction

Data were extracted by one author using electronic database software (SGS). Guided by the Cochrane Handbook for Systematic Reviews Handbook, two authors (SGS, IS) agreed on the appropriate variables to be extracted [19] and this was piloted by SGS. The variables extracted included study authors, date, location, design, analysis (qualitative), context (trial/non-trial), sample size, sample age, uptake levels, adherence levels, adherence type (day-to-day/persistence), factors tested for an association with adherence and qualitative themes.

### quality assessment

The Mixed Methods Appraisal Tool (MMAT) can be used to assess study quality in mixed study reviews [20]. The MMAT is reliable [21], and has been used in reviews of decision-making in the context of cancer [22, 23]. Each study is screened using two items related to the quality of the objectives, and the extent to which the data address the objectives. Study designs are classified as: (i) qualitative; (ii) quantitative randomized, controlled trials; (iii) quantitative non-randomized; (iv) quantitative descriptive; and (v) mixed methods. Study designs i–iv each have four of their own quality assessment items. Mixed methods studies are rated using three items, and then both sets of items for the two types of data reported (e.g. quantitative non-randomized and qualitative). All items are rated as ‘yes’, ‘no’ or ‘can't tell’, with one point awarded for each ‘yes’ response. Scores range from 0–4, with mixed method studies only able to score as highly as their lowest score for each study design. One researcher (SGS) assessed the quality of all included articles using the MMAT, and 20% of these were randomly selected and checked by a second researcher (AF) to ensure agreement. Discrepancies were resolved through discussion. MMAT scores were assessed at the study level and so were not necessarily associated with the quality of uptake and adherence data. To overcome this limitation, we created a single subjective evaluation assessing the extent to which the article contributed to our review.

### analysis

Random effect meta-analysis was used to allow for heterogeneity across uptake studies. Data were analysed in STATA 13.1 using the ‘metaprop’ command. Study heterogeneity was assessed with *Q* statistics and *I*^2^ estimations [24]. Results are plotted as a proportion (%) of women who have taken up preventive therapy with corresponding 95% confidence intervals and all *P*-values are two-sided. A quantitative synthesis of the adherence data was not possible due to differences in the data collection measure (e.g. pill count, clinical assessment, Medication Events Monitoring Systems) and type of adherence data collected (e.g. day-to-day, persistence or both). Therefore, a narrative synthesis describing these data was done. A narrative synthesis of the qualitative data was also carried out.

## results

**Figure 1.**
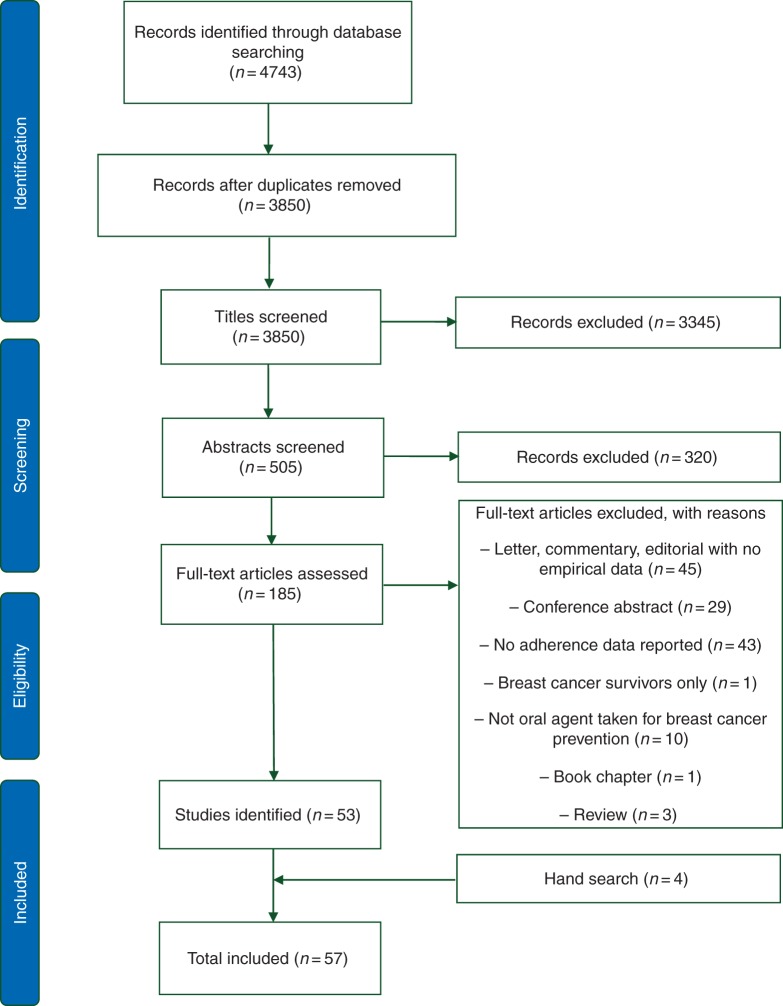
Flow diagram of search strategy.

The initial search yielded 4743 articles, of which 3850 remained after removing duplicates (Figure 1). Title screening led to 3345 exclusions, and a further 320 articles were removed after reviewing the remaining abstracts. One hundred and eighty-five full-text articles were assessed and 53 met inclusion/exclusion criteria. The reference lists of the remaining 53 articles were searched, and a further 4 manuscripts were identified. A total of 57 articles are included in the review.

### characteristics of included studies

Thirty-one articles reported uptake (Table 1) and 23 reported adherence (Table 2). Seventeen papers (30%) scored the maximum of 4/4 on the MMAT, the majority of which were non-randomized quantitative studies [26, 27, 35, 39, 41, 44, 47, 55, 56, 59, 61–64, 73, 75, 76]. Four studies (7%) met only one of the four assessment criteria [31, 37, 40, 67], all of which were randomized quantitative studies. Only three studies (5%) were given the highest rating of 4/4 using our subjective assessment [62, 63, 47], and five (9%) scored just 1/4 [57, 66, 71, 74, 77]. The mean quality score using the MMAT was 3.1 out of 4 compared with 2.5 out of 4 using the subjective assessment (supplementary Tables S1–S3, available at *Annals of Oncology* online).

**Table 1.** Characteristics of articles reporting uptake levels of breast cancer preventive therapy

Study	Country	Design	Setting	Agent	*n*	Age, years	Uptake
Altschuler and Somkin [25]	USA	Mixed	STAR trial	Tamoxifen; raloxifene	51	40–49 (2%); 50–59 (29%); 60–69 (35%); 70–79 (31%); >80 (2%)	54.9%
Bober et al. [26]	USA	Non-randomized	Non-trial; STAR	Tamoxifen; raloxifene	129	Mean, 52; SD, 8	25.6% (tamoxifen); 25.6% (STAR)
Collins et al. [27]	Australia	Non-randomized	kConFab	Tamoxifen	325	Median, 37, range 18–78	0.3% (tamoxifen); 2.8% (Trial)
Donnelly et al. [28]	UK	Mixed	Non-trial	Tamoxifen	1279	Median, 42	10.6%
Evans et al. [29]	UK	Non-randomized	IBIS1, IBIS2	Tamoxifen; anastrozole	2278; 1264	Not reported	12.0% (IBIS1); 8.1% (IBIS2)
Evans et al. [30]	UK	Non-randomized	IBIS1; LHRH	Tamoxifen; raloxifene	278; 142	Not reported	11.5% (IBIS1); 9.9% (LHRH)
Fagerlin et al. [31]	USA	Randomized	Non-trial	Tamoxifen; raloxifene	482	Mean, 62; SD, 5	0.4%
Goldenberg et al. [32]	USA	Non-randomized	Non-trial	Tamoxifen	99	Mean, 46	11.1%
Houlihan et al. [33]	USA	Non-randomized	STAR trial	Tamoxifen; raloxifene	242	Not described	33.5%
Juraskova et al. [34]	International	Randomized	IBIS2	Anastrozole	290	Mean, 59	46.4%
Yeomans Kinney et al. [35]	USA	Non-randomized	NSABP P-1	Tamoxifen	89	Mean, 59	43.8%
Yeomans-Kinney et al. [36]	USA	Non-randomized	NSABP P-1	Tamoxifen	175	Mean, 55; SD, 10	50.9%
Korfage et al. [37]	USA	Randomized	Non-trial	Tamoxifen; raloxifene	1012	Mean, 62; SD, 6	0.3%
Kwong et al. [38]	China	Non-randomized	Non-trial	Tamoxifen; raloxifene	26	Mean, 43; SD, 12	0%
Loehberg et al. [39]	Germany	Non-randomized	IBIS2	Anastrozole	2524	Mean 60; SD, 6	1.5%
Matloff et al. [40]	USA	Randomized	STAR trial	Tamoxifen; raloxifene	48	Mean, 49	0%
Metcalfe et al. [41]	International	Non-randomized	Non-trial	Tamoxifen; raloxifene	2677	Mean 46	5.5% (tamoxifen); 2.9% (raloxifene)
Metcalfe et al. [42]	International	Non-randomized	Non-trial	Tamoxifen; raloxifene	81	Mean, 45	12.3% (tamoxifen); 9.9% (raloxifene)
Ozanne et al. [43]	USA	Randomized	Non-trial	Tamoxifen; raloxifene	30	Control: mean, 44; SD, 10 versus Intervention: mean, 45; SD, 11	2/26 7.7%
Phillips et al. [44]	International	Non-randomized	kConFab	Tamoxifen	142	Mean, 41	0.7%
Port et al. [45]	USA	Non-randomized	Non-trial	Tamoxifen	43	Mean, 53	4.7%
Pujol et al. [46]	France	Non-randomized	LIBER	Letrozole	237	40–49 (36%), 50–69 (64%)	14.0%
Razzaboni et al. [47]	Italy	Non-randomized	IBIS II	Anastrozole	471	Mean, 59 (SD, 6)	29.1%
Rondanina et al. [48]	Italy	Non-randomized	HOT study	Tamoxifen	1457	Mean, 56 (SD, 5)	34.0%
Taylor and Taguchi [49]	Canada	Non-randomized	Non-trial	Tamoxifen; raloxifene	88	40–49 (12%), 50–59 (20%), 60–69 (37%), 70–80 (30%)	6.7%
Waters et al. [50]	USA	Non-randomized	NHIS survey	Tamoxifen	10 601; 10 690	40–79	0.2% (in 2000); 0.08% (in 2005)
Yeomans-Kinney et al. [51]	USA	Non-randomized	NSABP P-1	Tamoxifen	232	<50 (42%), 51+ (58%)	45.3%
Layeequr Rahman and Crawford [52]	USA	Non-randomized	Non-trial	Tamoxifen	48	Median 47; IQR, 42–53	31.3%
Metcalfe et al. [53]	Canada	Non-randomized	Non-trial	Tamoxifen; raloxifene	672	Mean, 47	6.3% (tamoxifen); 4.4% (raloxifene)
Tchou et al. [54]	USA	Non-randomized	Non-trial	Tamoxifen	219	Mean, 47	41.6%
Waters et al. [55]	USA	Non-randomized	NHIS survey	Tamoxifen; raloxifene	9906; 5959	35–79 (tamoxifen); 50–79 (raloxifene)	0.03% (2010; tamoxifen); 0.2% (raloxifene; 2010)

**Table 2.** Characteristics of articles reporting adherence data on breast cancer preventive therapy

Authors	Country	Design	Setting	Agent	*n*	Age (years)	Measure	Follow-up time (years)	Day-to-day adherence	Persistence
Cheung et al. [56]	International	Non-randomized	MAP.3	Exemestane	239	Median, 61; IQR, 59–65	Pill count	2	Median: 97%	–
Cuzick and Edwards [57]	International	Randomized	IBIS-1	Tamoxifen	4303	Not described	Pill count	1, 2, 4	–	90%; 83%; 74%
Cuzick et al. [58]	International	Randomized	IBIS-1	Tamoxifen	7154	Mean, 51	Pill count	5	–	67.9%
Day et al. [59]	USA	Non-randomized	NSABP P-1	Tamoxifen	11 064	Mean, 54; SD = 9	Clinic visit	3	–	80.8%
Day et al. [60]	USA	Non-randomized	NSABP P-1	Tamoxifen	11 064	Mean, 54; SD = 9	Clinic visit	3	–	69.1%
Fallowfield et al. [61]	UK	Non-randomized	IBIS1; TAMOPLAC	Tamoxifen	488	Median, 46	Self-report	5	–	61.8%
Juraskova et al. [34]	International	Randomized	IBIS2	Anastrozole	212	Mean, 59	Self-report	3 months	–	88.2%
Klepin et al. [62]	USA	Non-randomized	STAR trial	Tamoxife; raloxifene	1331	Mean, 67; SD, 4	Pill count	Unclear, probably 2	86.3%	–
Land et al. [63]	USA	Non-randomized	NSABP P-1	Tamoxifen	11 064	≥60 (30%)	Clinic visit	1 and 36 months	91%; 79%^a^	–
Land et al. [64]	USA	Non-randomized	STAR trial	Tamoxife; raloxifene	1983	35–49 (10%), 50–59: (49%); 60–69 (31%); 70+ (10%)	Clinic visit	5	–	Mean: 3 years
Maurice et al. [65]	UK	Non-randomized	IBIS1	Tamoxifen	82	Not described	MEMS	Adherence, 6 months; Persistence 5 years	Median % days correct dose: 93.2–95.2	79.3%
McTiernan et al. [66]	USA	Randomized	Trial	Aspirin	143	Mean, 60; SD, 6	Pill count	6 months	87%	–
Palva et al. [67]	Finland	Randomized	IBIS1	Tamoxifen	96	Placebo: mean, 50; SD, 8; Tamoxifen: mean, 51; SD, 8	Not reported	5	–	66.7%
Powles et al. [68]	UK	Randomized	Pilot	Tamoxifen	200	Tamoxifen: mean, 48; Placebo: mean, 49	Self-report	Months 3, 6, 9, 12	–	91.5%; 88.0%; 85.5%; 84.0%
Powles et al. [69]	UK	Randomized	Royal Marsden	Tamoxifen	2012	Median, 48	Self-report	5	–	80.8%
Powles et al. [70]	UK	Randomized	Royal Marsden	Tamoxifen	2471	Median, 47	Self-report	5	–	64.5%
Razzaboni et al. [47]	Italy	Non-randomized	IBIS II	Anastrozole	471	Mean, 59; SD, 6	Pill count	6 months, years 1, 2, 3	–	78.1%; 61.3%; 41.6%; 13.9%
Signori et al. [71]	USA	Randomized	Pilot	Raloxifene; omega-3 fatty acids	46	Mean, 56–58	Pill count	1	96%	–
Veronesi et al. [72]	Italy	Non-randomized	ITPS	Tamoxifen	201	Median, 53	Clinic visit	5	–	73.3%
Veronesi et al. [73]	International	Randomized	ITPS	Tamoxifen	3037	Median, 51	Clinic visit	1, 2, 3, 4, 5	–	86.1%; 80.1%; 76.2%; 74.2%; 73.7%
Vinayak et al. [74]	USA	Non-randomized	Trial	Lovastatin	30	Median, 45	Pill count	6 months	–	86.7%
Vogel et al. [75]	USA	Randomized	STAR trial	Tamoxife; raloxifene	19 471	Mean, 59; SD, 7	Not reported	4	–	68.3–71.5%
Vogel et al. [76]	USA	Randomized	STAR trial	Tamoxife; raloxifene	19 471	Mean, 59; SD, 7	Not reported	5	–	61.1–72.6%

RCT-SS, Randomized, controlled trial substudy.

^a^Reports a combined adherence and persistence measure; ITPS, Italian Tamoxifen Prevention Study.

Using MMAT categories, 34 studies used a non-randomized quantitative design [26, 27, 29, 30, 32, 33, 35, 36, 38, 39, 41, 42, 44–56, 59, 60–65, 72, 74], 16 used a randomized quantitative design [31, 34, 37, 40, 43, 57, 58, 66–71, 73, 75, 76], 5 studies were qualitative [77–81] and 2 were mixed-methods [25, 28]. Among the qualitative and mixed methods studies, five reported interview data [25, 28, 77, 79, 81] and two reported focus group data [78, 80]. The majority of quantitative studies (*n* = 36) were from trials [25, 29, 30, 33–36, 39, 40, 46–48, 51, 56–77], with 20 studies reporting non-trial data from clinics, cohorts and national surveys [27, 28, 31, 32, 37, 38, 41–45, 49, 50, 52–55, 78, 80, 81], and 2 studies included both trial and non-trial data [26, 79]. The majority of studies (*n* = 50) reported data on SERMs, with the remaining studies using aromatase inhibitors (AIs) (*n* = 6) [29, 34, 39, 46, 47, 56], aspirin [66], lovastatin [74] and luteinizing-hormone-releasing hormone (LHRH) [30].

The sample size of the quantitative studies ranged from 30 [43, 74] to 19 471 [75, 76], and the qualitative studies ranged from 2 [77] to 51 [25]. The studies were from a range of countries, including 30 from the USA [25, 26, 31–33, 35–37, 40, 43, 45, 50–55, 59, 60, 62, 64, 66, 71, 74, 75–78, 80, 81], 8 from the UK [28–30, 61, 65, 68–70], 3 from Italy [47, 48, 72], 3 from Canada [49, 53, 79] and 1 from each of Germany [39], Australia [27], China [38], France [46] and Finland [67]. Eight studies were international [34, 41, 42, 44, 56–58, 72]. Age was variably reported, but the lowest recorded was a median of 39 years [27] and the highest was a mean of 67 years [62].

### uptake of breast cancer preventive therapy

**Figure 2.**
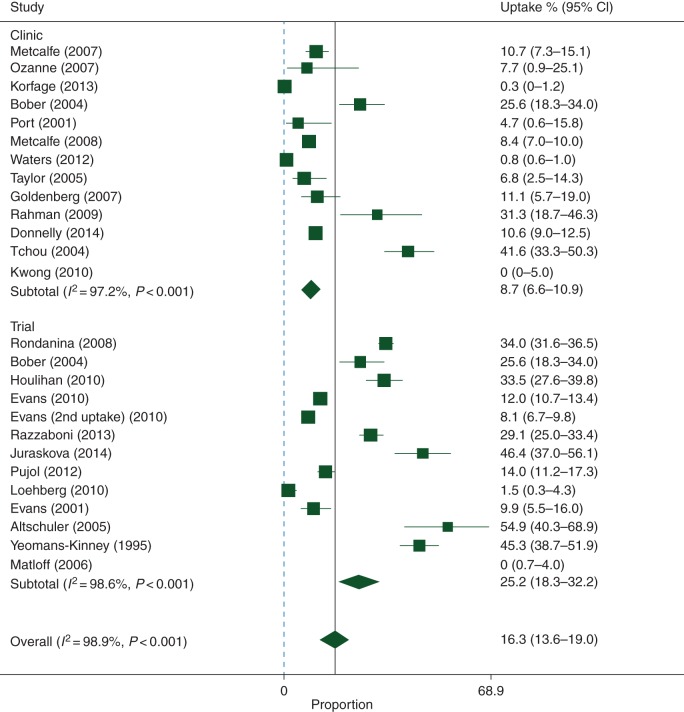
Meta-analysis of individual-level data for preventive therapy uptake by setting.

For the meta-analysis, 24 articles reporting 26 studies of uptake in 21 423 women were included. Seven articles reporting uptake were not included because more complete or similar data were available in another study [27, 31, 35, 36, 42, 44, 50]. Uptake ranged from 0% [38, 40] to 54.9% [25]. The pooled uptake estimate was 16.3% (95% CI 13.6–19.0), with high heterogeneity (*I*^2^ = 98.9%, *P* < 0.001) (Figure 2). Uptake was higher in trials [25.2% (95% CI 18.3–32.2)] than in non-trial settings [8.7% (95% CI 6.8–10.9)], and this difference was statistically significant (*P* < 0.001). Uptake was unaffected by agent and study location (supplementary Figures S1 and S2, available at *Annals of Oncology* online).

Fourteen of the uptake studies tested at least one predictor of uptake within the study (Table 3). Clinical factors associated with higher uptake in more than one study included having an abnormal breast biopsy [26, 54] and receiving a physician recommendation [26, 36]. Higher clinically assessed risk was associated with higher uptake in two studies [28, 54], but this effect was not consistent [36, 48]. Clinical factors reaching statistical significance in one study included having all questions answered by a physician, perceiving that the clinician supported their understanding of preventive therapy [48], and not having a BRCA mutation [28]. Previous experience of hot flashes was associated with lower uptake in one study [51], but there was no association in another [36]. There was no association between uptake and other clinical factors including the number of family members diagnosed [36, 47, 54], experiencing a breast biopsy [26, 54], previous hysterectomy [36, 51, 54] and menopausal status [51, 54].

**Table 3.** Summary of factors affecting uptake of breast cancer preventive therapy

	Bober et al. [26]	Donnelly et al. [28]	Evans et al. [29]	Goldenberg et al. [32]	Houlihan et al. [33]	Yeomans Kinney et al. [35]	Yeomans-Kinney et al. [36]	Metcalfe et al. [41]	Ozanne et al. [43]	Razzaboni et al. [47]	Rondanina et al. [48]	Yeomans-Kinney et al. [51]	Metcalfe et al. [53]	Tchou et al. [54]
Clinical factors														
Family member diagnosed							–			–				–
First-degree relative diagnosed	–													
First-degree relative died	–													
History breast biopsy	–													–
Abnormal breast biopsy	✓													✓
Family history of stroke	–													
Family history of cataracts	–													
Regular physician							–							
Physician recommendation	✓✓						✓✓							
Physician helped me understand											✓✓			
Physician answered all my questions											✓			
Having annual physical							–							
Objective risk		✓					–				–			✓
No BRCA mutation		✓												
Menopausal status												–		–
Hysterectomy							–					–		–
HRT/estrogen use^a^											X	✓		
Experience of hot flashes							–					✓		
Patient factors														
Concerned about side-effects^b^	✓					–						✓✓		
Concerned that estrogen contraindicated							✓✓					✓✓		
Believe that medication will not prevent cancer	✓						–							
Intrusive thinking	✓													
Depression	–										–			
Anxiety											–			
Life orientation	–													
Autonomy	–													
Knowledge of breast cancer							–							
Perceived risk (not described)							–							
Perceived risk (vulnerability)	✓													
Perceived risk (absolute)											–			
Perceived risk (relative)	✓										–			
Perceived risk (numerical)											–			
Worry about breast cancer											✓✓			
Peace of mind							–							
Concern about possibility of placebo						–	✓					✓✓		
Experimental nature of trial												✓		
Perceived expertise of clinician					–									
Personal desire to participate					✓✓									
Perceived value of trial					✓✓									
Perceived inconvenience of trial					✓✓									
Need to take a pill every day												–		
Frequency of clinic visits												✓		
Travel time to clinic												–		
Body mass index											–			
Smoking											–			
Alcohol consumption (low)											✓✓			
Physical activity											–			
Illegal drug use											–			
Prior use of screening							–							
Significant others reassured						–	✓							
Self-reported health							–							
Demographic factors														
Older age		✓					–			–	✓	X		✓
Race							–							
Country								✓						
Marital status						–				–		–		
Education							–			–	–	–		
Income												–		
Employment												–		
Insurance							–					–		
Cost						–	–					✓✓		
Parity														–

Notes: –, tested, but not statistically significant; ✓, tested in univariable analyses, and significant; ✓✓, tested multivariable, and significant; X, significant in opposite of hypothesized direction.

^a^Rondanina et al. [48] purposively sampled women who were currently taking or considering HRT for menopausal symptoms.

^b^Yeomans-Kinney et al. [51] tested multiple different concerns about side-effects, the results of which were mixed.

Lower uptake was consistently observed in women concerned about contradictions with estrogen [36, 51]. Greater concern about side-effects was associated with lower uptake in two studies [26, 51], although no relationship was found in another [35]. Statistically significant patient factors implicated in only one study included intrusive thinking [26], perceived vulnerability [26], worry about breast cancer [48], concern at the experimental nature of trials [51], personal desire to participate in a trial [33], perceived value of trials [33], perceived inconvenience of the trial [33], the frequency of clinic visits needed [51] and alcohol consumption [48]. There was mixed or no evidence for several other patient factors (Table 3).

No demographic factors were associated with uptake in more than one study. Country of residence was associated with uptake in a single study [41], with lower uptake in France, Italy, Holland and Norway. There was inconsistent or no evidence for age [28, 36, 47, 48, 51, 54], race [36], education [36, 47, 48, 51], income [51], employment status [35], insurance [36, 51], parity [54] and cost [35, 36, 51].

### adherence to breast cancer preventive therapy

All adherence studies were from trial data (Table 2). Studies investigating adherence mainly reported data on persistence (*n* = 18) [34, 47, 57–61, 64, 65, 67–70, 72–76]. Four reported data on day-to-day adherence [56, 65, 66, 71], and two used a hybrid measure of day-to-day adherence and persistence [62, 63]. Adherence measurement varied. Eight studies reported pill count data [47, 56–58, 62, 66, 71, 74], six noted adherence during a clinical visit [59, 60, 63, 64, 72, 73], five included self-report data [34, 61, 68–70], one used Medication Even Monitoring Systems (MEMS) [65] and three did not report how adherence was measured [67, 75, 76]. Eight studies reported data from a 5-year follow-up [58, 61, 64, 65, 67, 69, 70, 72, 73, 76], and the shortest end-point was 3 months [34].

Overall, studies suggested day-to-day adherence to preventive therapy was high, although all data were recorded within 2 years of initiating therapy. Day-to-day adherence was particularly high at 2-year follow-up in the MAP.3 exemestane trial (median, 97%) [56] and in a pilot trial of raloxifene with omega-3 followed up for 1 year (96%) [71]. A study using MEMS also suggested high rates of day-to-day adherence, at least in the first 6 months of therapy [65]. High rates of day-to-day adherence were reported over a 6-month period in an aspirin trial (87%) [66]. The two studies combining day-to-day adherence and persistence data reported high rates, although this was likely to decline over time [63]. One study only enrolled women who were adherent at baseline, which could bias subsequent reports [62].

Among studies reporting 5-year follow-up data, persistence ranged from 61.1% in the tamoxifen arm of the STAR trial [76] to 80.8% in both arms of the Royal Marsden trial [69]. However, a lower estimate of persistence (64.5%) in the Royal Marsden trial was reported elsewhere [70]. Several studies indicated adequate short-term persistence, which declined over time [57, 68, 73]. Italian data from the IBIS II Anastrozole trial reported a sharp decline in persistence from 78.1% at 6 months to 61.3%, 41.6% and 13.9% in years 1, 2 and 3 [47].

Eleven studies investigating either day-to-day adherence or persistence tested at least one predictor (Table 4). The most important clinical factor appeared to be the agent used. Five studies reported lower persistence to tamoxifen compared with placebo [61, 69, 70] and raloxifene [64, 67]. Two studies reported lower day-to-day adherence to tamoxifen compared with placebo [63] and raloxifene [62]. One study showed comparable persistence between tamoxifen and placebo [68], possibly due to low statistical power. Day-to-day adherence was similar between groups in a trial evaluating the effect of raloxifene versus placebo and versus omega-3 fatty acids [71]. Higher objective risk was associated with greater day-to-day adherence in one large study [63], although a smaller subsample of the IBIS 1 trial did not observe this effect [65]. Women with fewer depressive symptoms were more persistent in two studies [59, 62], but no effect was found in another [65]. There was mixed evidence for the relationship between persistence and use of other medications [62, 65]. There was no evidence for the remaining clinical factors (Table 4).

**Table 4.** Summary of factors affecting adherence to breast cancer preventive therapy

	Day et al. [59]	Fallowfield et al. [61]	Klepin et al. [62]	Land et al. [63]	Land et al. [64]	Maurice et al. [65]	Palva et al. [67]	Powles et al. [68]	Powles et al. [69]	Powles et al. [70]	Signori et al. [71]
Clinical factors											
Placebo versus tamoxifen (tamoxifen lower)		✓		✓✓				–	✓	✓	
Raloxifene versus tamoxifen (tamoxifen lower)			✓✓		✓		✓				
Higher objective risk				✓✓		–					
Presence of diabetes			–								
Presence of heart disease			–								
Presence of impaired vision			–								
Less depression	✓		✓			–					
Diagnosis of prior malignancy			–								
Comorbid condition				–							
Taking other medications			–			✓					
Hysterectomy						–					
Menopausal status						–	–				
Previous breast biopsy						–					
Patient factors											
Longer expected time on treatment			✓✓								
Cognitive ability^a^			–								
Alcohol consumption				–							
Non-smoker				✓✓		✓					
Overweight/obese				–							
Physical activity				–							
Demographic factors											
Younger age			✓	✓✓		–					
Ethnicity			–	–							
More education			–	✓✓							
Employment				–							
Income				–							
Living alone				–							
Marital status						–					
Parity						–					

Notes: –, tested, but not statistically significant; ✓, tested in univariable analyses, and significant; ✓✓, tested in multivariable analyses, and significant.

^a^Keplin et al. tested multiple different cognitive abilities and only verbal fluency (✓✓) and verbal fluency were significant (✓✓).

Non-smoking status was linked with higher day-to-day adherence in two studies [63, 65]. One study suggested participants who expected to be on therapy for longer were more adherent [62]. The same study also demonstrated greater day-to-day adherence among those with higher verbal memory, although multiple other cognitive domains were tested which showed no effect [62]. There was no evidence for a relationship between adherence and alcohol consumption [63], overweight [63] and physical activity [63]. No demographic factor was consistently associated with adherence, although two large studies suggested younger age was linked with higher day-to-day adherence [62, 63], and one suggested higher levels among the more educated [63]. There was no evidence of other socioeconomic disparities, as assessed by ethnicity [62, 63], employment [63] or income [63]. There was also no relationship between day-to-day adherence and living alone [63], marital status [65] or parity [65].

A relationship between side-effects and adherence was suggested by reports of lower persistence among women taking tamoxifen compared with placebo and raloxifene [61–64, 67, 69, 70]. However, the quality of side-effect assessment was poor. The primary tool for assessment was ‘off-therapy forms’ (OTFs) provided only to women who did not persist with the medication. These data are likely to be subject to attribution bias. Seven tamoxifen studies used OTFs to document the proportion of women who attributed their drop-outs to side-effects [59, 60, 63, 67–70] and one anastrozole trial used an OTF [47]. Data from three placebo-controlled trials reported a higher proportion of side-effect-related drop-outs among women taking tamoxifen [60, 67, 70], although almost half of the women stopping prematurely attributed their decision to non-medical factors [60, 70].

### qualitative data on breast cancer preventive therapy decision-making

The characteristics of the qualitative studies are shown in Table 5 and the extracted themes are presented in Table 6. All seven qualitative studies included were related to women's attitude towards tamoxifen or raloxifene, and their decision to initiate preventive therapy. All studies discussed at least one aspect of breast cancer risk. Five studies reported that women with a heightened perceived personal risk were more likely to use preventive therapy [25, 78–81], with low perceived risk resulting from a sense of wellness [78] or lack of symptoms [81]. Taking preventive therapy was considered to be a daily reminder of one's risk [28], which some women preferred to deny [79] or seek alternative strategies [80]. A Canadian study noted unrealistic views about prevention among some women, with risk-reduction expectations ranging from 50% to 100% [79]. Three studies reported that concerns about side-effects were a deterrent to uptake [25, 28, 79]. One diverse focus group study noted a low awareness of preventive therapy [78], which may be as a result of a lack of information about the topic [79] and poor patient–provider communication [78]. Two other studies reported a low level of understanding regarding the causes of breast cancer [78, 81]. The use of medication for prevention was considered to be an important topic [81], with women reporting concerns about drug interactions [78], the ‘unnatural’ nature of medications [78, 79, 81] and worries that HRT would be contraindicated [79, 25]. One high-quality study reported women were reluctant to use tamoxifen because they considered it to be a ‘cancer drug’ that was inextricably linked with the disease and their family's history of using the drug [28]. Several trial-related factors were barriers to enrolment including the time commitment and the concept of randomization [25]. Altruism was a motivating factor for some women [25, 79]. Factors mentioned in only one study can be found in Table 6.

**Table 5.** Characteristics of qualitative studies discussing breast cancer preventive therapy decision-making

Study	Country	Design	Analysis	Setting	Agent	*n*	Age, years (% of sample)
Altschuler and Somkin [25]	USA	Mixed	Grounded theory	STAR	Tamoxifen; raloxifene	51	40–49 (2%); 50–59 (29%); 60–69 (35%); 70–79 (31%); >80 (2%)
Cyrus-David and Strom [78]	USA	Qualitative	Cross-case analysis using variable-oriented strategies	Non-trial	Tamoxifen; raloxifene	26	30–59 (54%); ≥60 (42%); unknown (4%)
Donnelly et al. [28]	UK	Mixed	Framework analysis	Non-trial	Tamoxifen	30	Median, 42
Heisey et al. [79]	Canada	Qualitative	Framework analysis	Non-trial; STAR	Tamoxifen; raloxifene	27	Median, 61
Holmberg et al. [77]	USA	Qualitative	Narrative theory	STAR	Tamoxifen	2	73 and 52
Paterniti et al. [80]	USA	Qualitative	Unclear, likely to be thematic	Non-trial	Tamoxifen	27	68.3 years (61–78)
Salant et al. [81]	USA	Qualitative	Grounded theory	Non-trial	Tamoxifen	33	Mean 55 (range, 33–70)

**Table 6.** Qualitative themes affecting decision-making and uptake of preventive therapy

	Risk	Side-effects	Knowledge	Medication concerns	Information	Trial- issues	Other
Altschuler and Somkin [25]	Perceived personal risk; threat of other disease	Side-effect concerns		Concern about contraindication of HRT		Altruism; time; commitment; randomization	
Cyrus-David et al. [78]	Accuracy of risk perceptions; perceived wellness		Knowledge of risk factors; awareness of chemoprevention	Drug interactions; chemical properties of drugs; length of treatment	Patient–provider communication		Distrust of medical system; conception issues; cost
Donnelly et al. [28]	Daily reminder of risk	Side-effect concerns		Tamoxifen as a ‘cancer drug’			Impact of others' experience
Heisey et al. [79]	Perceived personal risk; denial of risk; expectations for risk-reduction	Side-effect concerns		Aversion to medication; HRT controversies	Lack of information; information sources	Altruism	Being in control; term ‘chemoprevention’; cost
Holmberg et al. [77]	The meaning of ‘risk’; personalized risk assessments; concern about possible diagnosis; comparisons with coronary heart risk						
Paterniti et al. [80]	Perceived personal risk; alternative approaches to reducing risk			Risks and benefits of tamoxifen			Meaning of breast cancer; religiosity
Salant et al. [81]	Perceived personal risk; lack of symptoms/problems		Mythical causes of breast cancer	Dislike of medication; use of medication to treat rather than prevent			Cognitive avoidance of cancer

## discussion

In this systematic review of studies investigating decision-making in the context of breast cancer preventive therapy, we observed low uptake of all agents and poor long-term persistence. In our meta-analysis including over 21 000 women, only one in six women decided to take preventive therapy or enter a chemoprevention trial. We were unable to explain the heterogeneity observed in the model using pre-specified subgroup analyses comparing agent, context and location. Short-term persistence was high, and women demonstrated adequate use of medications on a day-to-day basis. However, persistence with preventive therapy for 5 years was low, limiting the preventive effect in these women. These data suggest future research should be directed towards supporting decision-making at the point of uptake, as well as ensuring mechanisms are in place to promote persistence among women who have initiated therapy.

Our estimate of uptake is comparable with a previous meta-analysis reporting 15% of women accepted the offer of preventive therapy in five studies outside a trial setting [14]. However, subgroup analysis suggested uptake in clinical settings was significantly lower than this estimate. The difference in uptake between settings suggests issues with implementing preventive therapy within routine patient care. Clinician's attitudes towards the topic of preventive therapy are not well known, but prescribing concerns may affect their willingness to discuss this option [82]. For example, tamoxifen and raloxifene are not licensed for prevention in some countries, which can dissuade prescribing [82–84]. Discussing medication and writing prescriptions are also unfamiliar tasks for many clinicians working with high-risk populations. Providing appropriate support and training may encourage the implementation of preventive therapy into routine patient care.

There was considerable heterogeneity in our uptake estimate, and this is likely to be a result of specific studies reporting high enrolment rates. The highest uptake (54.9%) was reported in a small (*n* = 51) mixed methods study, where interest may have been higher because the study protocol involved attendance at an interview [25]. Similarly, uptake in specific centres of the IBIS-II trial was high, perhaps because enrolment was only discussed with women actively seeking information about the trial [34]. Caution should therefore be taken when interpreting these uptake data, as they may include populations who are more interested in prevention than the general population. They also only include women who have actively sought clinician advice about their breast cancer risk. Other clinical groups such as those with benign breast disease [85], dense breasts [13] and older women may meet risk thresholds, but are not routinely offered preventive therapy.

Efforts to support patient decision-making may be guided by our attempt to identify the factors related to higher uptake and adherence. Concerns about medication were important in both quantitative and qualitative studies within this review. For example, in a US study of 129 women with follow-up at 2 and 4 months after counselling, those who were more concerned about side-effects or were unconvinced by tamoxifen's preventive effect were less likely to initiate therapy [26]. Other concerns included the perception that tamoxifen was a ‘cancer drug’ that would serve as a reminder of family members who had used it [28]. Mistrust of medication in general was also a common attitude [79, 81]. These observations support a meta-analysis of the Necessity Concerns Framework, which showed lower adherence among patients who felt medication was an unnecessary part of their disease management, or among those who expressed greater concerns about the use of medication [86]. Attempts to correct such beliefs have had mixed results [87–89], but several studies have indicated that necessity beliefs and concerns are amenable to change [90–92].

Data from our review suggest receipt of a clinician recommendation may not be sufficient to increase uptake [26, 36], but discussions about the risks and benefits of preventive therapy are necessary for informed decision-making [93]. Studies suggested women making informed decisions were equally likely to initiate therapy. One study reported higher uptake among patients who believed that all their questions had been answered and that their clinician had helped them understand [48]. A decision-aid tested in the context of a clinical trial was also effective in supporting women's decision-making, without reducing uptake [34]. There is a clear demand for information about preventive therapy [79], and awareness levels are low [78]. Women's decision-making about preventive therapy could benefit from patient-centred communications, which outline the risks and benefits of preventive therapy in a comprehensible manner [94].

Studies comparing tamoxifen with placebo or raloxifene consistently reported higher drop-out rates among the tamoxifen arm, suggesting side-effects unique to the drug may be responsible [61–64, 67, 69, 70]. Furthermore, several studies collecting OTFs suggested over half of all drop-outs were a result of medication side-effects [59, 60, 63, 67–70]. Clinicians counselling women with side-effects from tamoxifen could consider prescribing more tolerable agents with similar effectiveness [75, 76]. While these data are somewhat useful in explaining low long-term persistence, the method is likely to be prone to bias. For example, women who had already chosen to cease participation may have been more likely to attribute their decision to a medical factor, thereby exaggerating the importance of side-effects. To resolve this issue, future studies are needed that prospectively collect patient-reported outcome data to enable comparisons between those who do and do not persist. In the meantime, accurate side-effect data should be conveyed to women who express concerns about safety [26, 51, 78, 79, 81].

Due to differences in the reporting and recording of adherence, we were unable to synthesize the data in a meta-analysis. Despite advantages and disadvantages to different methods, there is currently no gold standard for defining or measuring adherence. This is a limitation in all settings in which medication is taken, and is not solely observed in oncology. Research is needed that not only seeks ways to promote adherence to these therapies, but more broadly can standardize the manner in which this behaviour is quantitatively assessed to allow a better comparison between studies. This would include agreed upon means for classifying adherence, including evidence-based thresholds for what can be considered adequate adherence. The review was further limited by the low number of studies included in countries outside of the USA and Europe. This should be addressed in the light of the rising incidence rates in developing countries [7]. There were also insufficient reports of agents other than SERMs. The ongoing evaluation of next-generation agents such as AIs should be accompanied by detailed adherence reports.

## conclusions

Preventive therapy uptake for the prevention of breast cancer is low, and long-term persistence is often insufficient for women to experience the full preventive effect. Uptake is higher in trial settings, suggesting further work is needed to identify the problems with implementing preventive therapy within routine clinical practice. Improving the communication of information about preventive therapy is likely to benefit women, but further research should identify additional factors amendable to modification to promote informed decisions related to chemoprevention.

## funding

SGS is supported by a Cancer Research UK Postdoctoral Fellowship (C42785/A17965). AF is supported by a Cancer Research UK—BUPA Cancer Prevention Postdoctoral Fellowship (C49896/A17429). RH is supported by NIHR Collaboration for Leadership in Applied Research and Care (CLAHRC) North Thames. The sponsor of the study played no role in the design, collection, analysis, interpretation of the data, writing of the manuscript or decision to submit the manuscript for publication.

## disclosure

JC received research funds from AstraZeneca to undertake the IBIS studies. JC has no financial ties with them. All remaining authors declare no conflict of interest.

## Supplementary Material

Supplementary Data
