# Supplementary material for: Factors affecting uptake and adherence to breast cancer chemoprevention: a systematic review and meta-analysis
Source: Ann Oncol. 2015 Dec 8;27(4):575–90. doi: 10.1093/annonc/mdv590 (PMC4803450; doi:10.1093/annonc/mdv590)
Supplement: Supplementary Data [file supp_27_4_575__index.html]

Factors affecting uptake and adherence to breast cancer chemoprevention: a systematic review and meta-analysis — Supplementary Data 

# Factors affecting uptake and adherence to breast cancer chemoprevention: a systematic review and meta-analysis

## Supplementary Data

Supplementary Data

- Supplementary Checklist - doc file
- Supplementary Search Terms - docx file
- Supplementary Figure 1 - docx file
- Supplementary Figure 2 - docx file
- Supplementary Table 1 - docx file
- Supplementary Table 2 - docx file
- Supplementary Table 3 - docx file
