# Supplementary material for: Factors affecting uptake and adherence to breast cancer chemoprevention: a systematic review and meta-analysis
Source: Ann Oncol. 2015 Dec 8;27(4):575–90. doi: 10.1093/annonc/mdv590 (PMC4803450; doi:10.1093/annonc/mdv590)
Supplement: Supplementary Data [file supp_mdv590_mdv590supp_searchterms.docx]

PubMed search terms

((((((((((((((((((((((Pharmacoadherence) OR therapeutic alliance) OR pharmionics) OR concordance) OR persistenc*) OR compliance) OR choos*) OR decid*) OR participat*) OR choice*) OR decision-making) OR decision) OR enrol*) OR initiat*) OR uptake) OR adher*) OR medication adherence) OR nonadherence) OR non-adherence) OR discontinu*)) AND ((((((((((((((((((((((chemoprevent*) OR antineoplastic agents) OR Selective Oestrogen Receptor Modulators) OR Aromatase Inhibitors) OR hormonal agents) OR Bisphosphonates) OR Metformin) OR Non-steroidal anti-inflammatory drugs) OR COX-2 inhibitors) OR Aspirin) OR tamoxifen) OR raloxifene) OR lasofoxifene) OR arzoxifene) OR Anastrozole) OR Exemestane) OR Fenretinide) OR Tibolone) OR statins) OR ibuprofen) OR letrozole) OR vitamin D)) AND ((((prophylactic) OR prevent*)) AND breast cancer)
