# Supplementary material for: Factors affecting uptake and adherence to breast cancer chemoprevention: a systematic review and meta-analysis
Source: Ann Oncol. 2015 Dec 8;27(4):575–90. doi: 10.1093/annonc/mdv590 (PMC4803450; doi:10.1093/annonc/mdv590)

Supplementary Figure 2. Meta-analysis of individual-level data for preventive therapy uptake by location


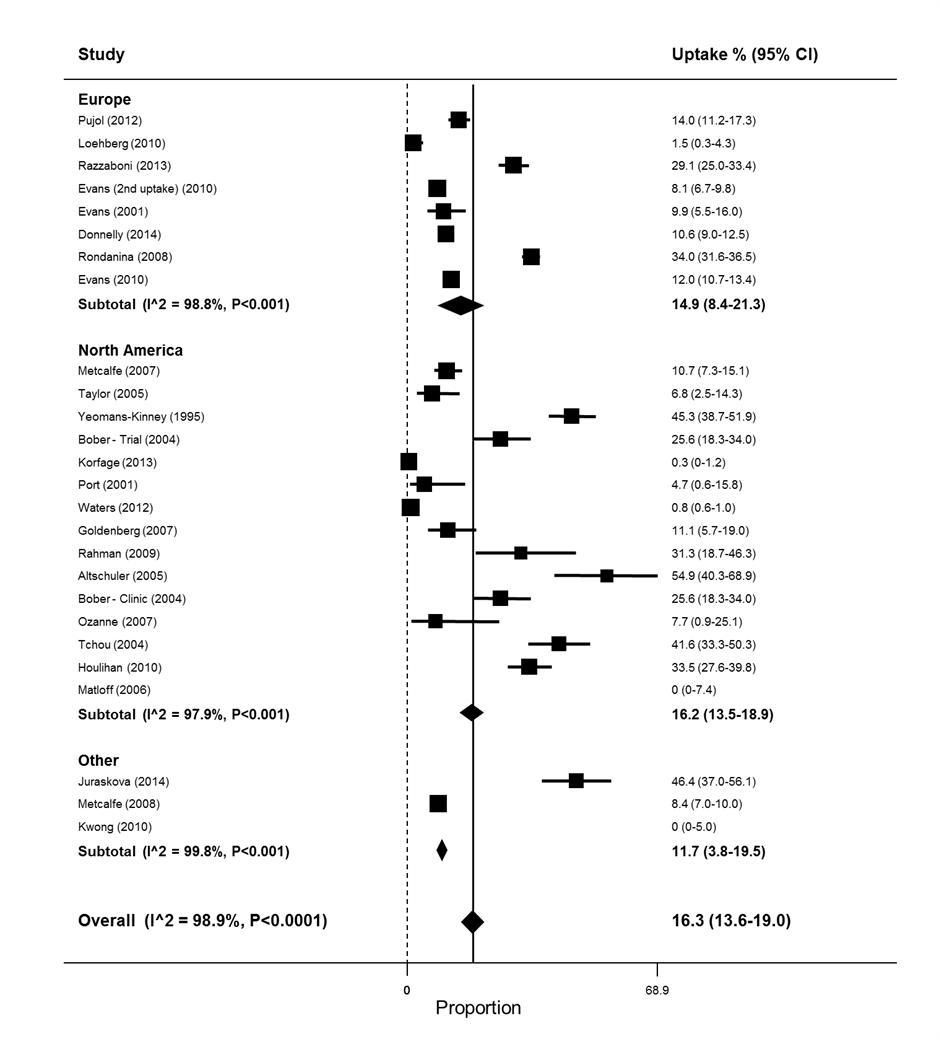

Supplement: Supplementary Data [file supp_mdv590_mdv590supp_fig2.docx]
