# Supplementary material for: Factors affecting uptake and adherence to breast cancer chemoprevention: a systematic review and meta-analysis
Source: Ann Oncol. 2015 Dec 8;27(4):575–90. doi: 10.1093/annonc/mdv590 (PMC4803450; doi:10.1093/annonc/mdv590)
Supplement: Supplementary Data [file supp_mdv590_mdv590supp_table1.docx]

| Supplementary Table 1. Qualitative study quality assessment using the Mixed Methods Appraisal Tool and researcher assessment | | | | | | | | | |
| --- | --- | --- | --- | --- | --- | --- | --- | --- | --- |
| Authors and date | Type of study | Is there a clear research question? | Do the data address the research question? | Are the data sources relevant to the research question? | Is analysis relevant to address the research question? | Is consideration given to how findings related to the context? | Is appropriate consideration given to how findings related to researchers’ influence? | Overall MMAT score | Assessment of contribution to review |
| Altschuler et al., 2005 | Mixed | Yes | Yes | Yes | Yes | Yes | No | *** | ** |
| Cyrus-David et al., 2001 | Qualitative | Yes | Yes | Yes | Yes | No | No | ** | ** |
| Donnelley et al., 2014 | Mixed | Yes | Yes | Yes | Yes | Yes | No | *** | *** |
| Heisey et al., 2006 | Qualitative | Yes | Yes | Yes | Yes | Yes | No | *** | ** |
| Holmberg et al., 2010 | Qualitative | Yes | No | Yes | Yes | No | No | ** | * |
| Paterniti et al., 2005 | Qualitative | Yes | Yes | Yes | Yes | Yes | No | *** | ** |
| Salant et al., 2006 | Qualitative | Yes | Yes | Yes | Yes | Yes | No | *** | ** |

Note: * rating is out of 4 for both MMAT score and reviewer assessment
