# Supplementary material for: Factors affecting uptake and adherence to breast cancer chemoprevention: a systematic review and meta-analysis
Source: Ann Oncol. 2015 Dec 8;27(4):575–90. doi: 10.1093/annonc/mdv590 (PMC4803450; doi:10.1093/annonc/mdv590)
Supplement: Supplementary Data [file supp_mdv590_mdv590supp_table2.docx]

| Supplementary Table 2. Randomized quantitative study quality assessment using the Mixed Methods Appraisal Tool and researcher assessment | | | | | | | | | |
| --- | --- | --- | --- | --- | --- | --- | --- | --- | --- |
| Authors and date | Type of study | Is there a clear research question? | Do the data address the research question? | Is there a clear description of the randomisation? | Is there a clear description of the allocation concealment? | Are there complete outcome data? (>80%) | Is there low withdrawal / drop out (<20%) | Overall MMAT score | Assessment of contribution to review |
| Cuzick & Edwards, 1999 | Randomized | Yes | Yes | Can't tell | Can’t tell | Yes | Yes | ** | * |
| Cuzick et al., 2007 | Randomized | Yes | Yes | Yes | Yes | Yes | Yes | ** | ** |
| Fagerlin et al., 2011 | Randomized | Yes | Yes | Can't tell | Can't tell | Yes | No | * | *** |
| Juraskova et al., 2014 | Randomized | Yes | Yes | Yes | Yes | Yes | No | *** | *** |
| Korfage et al., 2013 | Randomized | Yes | Yes | Can't tell | Can't tell | Yes | No | * | *** |
| Matloff et al., 2006 | Randomized | Yes | Yes | No | Can't tell | Yes | No | * | ** |
| McTiernan et al., 2009 | Randomized | Yes | Yes | Can't tell | Yes | Yes | Yes | *** | * |
| Ozanne et al,. 2007 | Randomized | Yes | Yes | Can't tell | Can't tell | Yes | Yes | ** | ** |
| Palva et al., 2013 | Randomized | Yes | Yes | Can't tell | Yes | No | No | * | ** |
| Powles et al., 1989 | Randomized | Yes | Yes | Can't tell | Yes | Yes | Yes | *** | ** |
| Powles et al. 1994 | Randomized | Yes | Yes | Can't tell | Yes | Yes | Yes | *** | ** |
| Powles et al. 1998 | Randomized | Yes | Yes | Can't tell | Yes | Yes | Yes | *** | ** |
| Signori et al., 2012 | Randomized | Yes | Yes | Yes | Can't tell | Yes | Yes | *** | * |
| Veronesi et al., 1998 | Randomized | Yes | Yes | Yes | Yes | Yes | Yes | **** | ** |
| Vogel et al., 2006 | Randomized | Yes | Yes | Yes | Yes | Yes | Yes | **** | ** |
| Vogel et al., 2010 | Randomized | Yes | Yes | Yes | Yes | Yes | Yes | **** | ** |

Note: * rating is out of 4 for both MMAT score and reviewer assessment
