# Supplementary material for: Factors affecting uptake and adherence to breast cancer chemoprevention: a systematic review and meta-analysis
Source: Ann Oncol. 2015 Dec 8;27(4):575–90. doi: 10.1093/annonc/mdv590 (PMC4803450; doi:10.1093/annonc/mdv590)
Supplement: Supplementary Data [file supp_mdv590_mdv590supp_table3.docx]

| Supplementary Table 3. Non-randomized quantitative study quality assessment using the Mixed Methods Appraisal Tool and researcher assessment | | | | | | | | | |
| --- | --- | --- | --- | --- | --- | --- | --- | --- | --- |
| Authors and date | Type of study | Is there a clear research question? | Do the data address the research question? | Are participants recruited in a way that minimizes bias? | Are measurements appropriate? | Are groups comparable / differences controlled? | Are there complete data and response rates? | Overall MMAT score | Assessment of contribution to review |
| Altschuler et al., 2005 | Mixed | Yes | Yes | Yes | Yes | Can't tell | Yes | *** | ** |
| Bober et al., 2004 | Non-randomized | Yes | Yes | Yes | Yes | Yes | Yes | **** | *** |
| Cheung et al., 2012 | Non-randomized | Yes | Yes | Yes | Yes | Yes | Yes | **** | ** |
| Collins et al., 2013 | Non-randomized | Yes | Yes | Yes | Yes | No | Yes | *** | ** |
| Day et al., 2001 | Non-randomized | Yes | Yes | Yes | Yes | Yes | Yes | **** | *** |
| Day et al., 1999 | Non-randomized | Yes | Yes | Yes | Yes | Yes | Yes | **** | ** |
| Donnelley et al., 2014 | Mixed | Yes | Yes | Yes | Yes | Yes | Yes | *** | *** |
| Evans et al., 2010 | Non-randomized | Yes | Yes | Yes | Yes | Can't tell | Yes | *** | *** |
| Evans et al., 2001 | Non-randomized | Yes | Yes | Yes | Yes | Can't tell | Yes | *** | *** |
| Fallowfield et al., 2001 | Non-randomized | Yes | Yes | Yes | Yes | Yes | Yes | **** | *** |
| Goldenberg et al., 2007 | Non-randomized | Yes | Yes | Yes | Yes | No | Yes | *** | *** |
| Houlihan et al., 2010 | Non-randomized | Yes | Yes | Yes | Yes | Yes | No | *** | *** |
| Kinney et al., 1998 | Non-randomized | Yes | Yes | No | Yes | Yes | Yes | *** | *** |
| Kinney et al., 1998 | Non-randomized | Yes | Yes | Yes | Yes | Yes | Yes | **** | *** |
| Klepin et al., 2014 | Non-randomized | Yes | Yes | Yes | Yes | Yes | Yes | **** | **** |
| Kwong et al., 2010 | Non-randomized | Yes | Yes | Yes | Yes | No | Yes | *** | ** |
| Land et al., 2011 | Non-randomized | Yes | Yes | Yes | Yes | Yes | Yes | **** | **** |
| Land et al,. 2006 | Non-randomized | Yes | Yes | Yes | Yes | Yes | Yes | **** | *** |
| Loehberg et al., 2010 | Non-randomized | Yes | Yes | Yes | Yes | Yes | Yes | **** | ** |
| Maurice et al., 2006 | Non-randomized | Yes | Yes | Yes | Yes | No | Yes | *** | *** |
| Metcalfe et al., 2008 | Non-randomized | Yes | Yes | Yes | Yes | Yes | Yes | **** | *** |
| Metcalfe et al., 2005 | Non-randomized | Yes | Yes | Yes | Yes | No | Yes | *** | ** |
| Phillips et al., 2006 | Non-randomized | Yes | Yes | Yes | Yes | Yes | Yes | **** | ** |
| Port et al., 2001 | Non-randomized | Yes | Yes | Yes | Yes | Can't tell | Yes | *** | ** |
| Pujol et al., 2012 | Non-randomized | Yes | Yes | Can't tell | Yes | No | Yes | ** | ** |
| Razzaboni et al., 2013 | Non-randomized | Yes | Yes | Yes | Yes | Yes | Yes | **** | **** |
| Rondanina et al., 2008 | Non-randomized | Yes | Yes | Yes | Yes | No | No | ** | *** |
| Taylor & Taguchi, 2005 | Non-randomized | Yes | Yes | Can't tell | Yes | Can't tell | Yes | ** | ** |
| Veronesi et al., 1998 | Non-randomized | Yes | Yes | Yes | Yes | Can’t tell | Yes | *** | ** |
| Vinayak et al., 2013 | Non-randomized | Yes | Yes | No | Yes | Can't tell | Yes | ** | * |
| Waters et al., 2010 | Non-randomized | Yes | Yes | Yes | Yes | Yes | Yes | *** | *** |
| Yeomans-Kinney et al., 1995 | Non-randomized | Yes | Yes | No | Yes | Yes | Yes | *** | *** |
| Rahman & Crawford, 2009 | Non-randomized | Yes | Yes | Can't tell | Yes | Can't tell | Yes | ** | ** |
| Metcalfe et al., 2007 | Non-randomized | Yes | Yes | Yes | Yes | Can't tell | Can't tell | ** | ** |
| Tchou et al., 2004 | Non-randomized | Yes | Yes | Yes | Yes | No | Yes | *** | ** |
| Waters et al., 2012 | Non-randomized | Yes | Yes | Yes | Yes | Yes | Yes | **** | *** |

Note: * rating is out of 4 for both MMAT score and reviewer assessment
